# A System of Anatomy for the Use of Students of Medicine

**Published:** 1843-04

**Authors:** 


ART. IV.—*A System of Anatomy for the use of Students of Medicine.* By CASPAR WISTAR, M. D., late Professor of Anatomy in the University of Pennsylvania. *With notes and additions.* By WILLIAM E. HORNER, M. D., Professor of Anatomy in the University of Pennsylvania. Eighth edition. *Entirely remodelled, and Illustrated by more than two hundred engravings.* By J. PANCOAST, M. D., Professor of General, Descriptive and Surgical Anatomy in Jefferson Medical College, etc. 2 vols., 8vo., pp. 538, 622. Philadelphia: Thomas, Cowperthwait & Co.; 1842.

Dr. Wistar's Anatomy has always been a favorite with students, and has accordingly passed through numerous editions. Indeed it has been *worked over* so often, that we can scarcely recognize it as the book with which we began our professional studies; even at that time it had been more or less changed by editors.

Professor Pancoast has retained the matter introduced by the former editor, Professor Horner, to which he has added a large amount of new matter, compiled from various sources, chiefly in relation to general and microscopic anatomy, the latter of which has been for some time and is now attracting so much attention. The work is fully brought up to the existing state of the science. It is embellished by the introduction of colored copperplates, eight in number, from Bell, and upwards of two hundred wood-cuts, selected from various authors. The latter are executed in superior style, as are all other portions of the work. In truth, of the many reprints that have come under our notice, this is by far the most beautiful in appearance. It cannot fail we should think to be received with equal favor as the former editions. C.

